# The relationship between parental depressive symptoms and offspring psychopathology: evidence from a children-of-twins study and an adoption study

**DOI:** 10.1017/S0033291715000501

**Published:** 2015-05-21

**Authors:** T. A. McAdams, F. V. Rijsdijk, J. M. Neiderhiser, J. Narusyte, D. S. Shaw, M. N. Natsuaki, E. L. Spotts, J. M. Ganiban, David Reiss, L. D. Leve, P. Lichtenstein, T. C. Eley

**Affiliations:** 1 MRC Social, Genetic and Developmental Psychiatry Centre, Institute of Psychiatry, Psychology and Neuroscience, King's College London, London, UK; 2 Department of Psychology, Penn State University, USA; 3 Department of Clinical Neusroscience, Karolinska Institutet, Stockholm, Sweden; 4 Department of Psychology, University of Pittsburgh, USA; 5 Department of Psychology, University of California Riverside, USA; 6 Office of Behavioral and Social Science Research, NIH, Bethesda, MD, USA; 7 Department of Psychology, George Washington University, Washington, DC, USA; 8 Child Study Center, Yale University, New Haven, CT, USA; 9 Department of Counseling Psychology and Human Services, University of Oregon, and Oregon Social Learning Center, Eugene, Oregon, USA; 10 Department of Medical Epidemiology and Biostatistics, Karolinksa Institute, Stockholm, Sweden

**Keywords:** Adoption, children of twins, externalizing, gene–environment correlation, internalizing, parental depression

## Abstract

**Background:**

Parental depressive symptoms are associated with emotional and behavioural problems in offspring. However, genetically informative studies are needed to distinguish potential causal effects from genetic confounds, and longitudinal studies are required to distinguish parent-to-child effects from child-to-parent effects.

**Method:**

We conducted cross-sectional analyses on a sample of Swedish twins and their adolescent offspring (*n* = 876 twin families), and longitudinal analyses on a US sample of children adopted at birth, their adoptive parents, and their birth mothers (*n* = 361 adoptive families). Depressive symptoms were measured in parents, and externalizing and internalizing problems measured in offspring. Structural equation models were fitted to the data.

**Results:**

Results of model fitting suggest that associations between parental depressive symptoms and offspring internalizing and externalizing problems remain after accounting for genes shared between parent and child. Genetic transmission was not evident in the twin study but was evident in the adoption study. In the longitudinal adoption study child-to-parent effects were evident.

**Conclusions:**

We interpret the results as demonstrating that associations between parental depressive symptoms and offspring emotional and behavioural problems are not solely attributable to shared genes, and that bidirectional effects may be present in intergenerational associations.

## Introduction

Parental depression correlates with offspring emotional and behavioural problems (Lovejoy *et al.* 2000; Kane & Garber, 2004; Barker *et al.* 2012). This has been reported in studies using categorical measures to identify depressed parents (Fendrich *et al.* 1990; Lieb *et al.* 2002), and continuous measures of depressive symptoms (Davies & Windle, 1997; Cummings *et al.* 2005). Such findings indicate the presence of one or more of three mechanisms: First, exposure to parental depressive symptoms may play a role in the development of offspring psychopathology. This could occur via negative parenting practices associated with depression (Kendler, 1996; Burt *et al.* 2005; Elgar *et al.* 2007), or via learning processes such as imitation or modelling (Bandura, 1977). Second, exposure to offspring psychopathology may increase parental depressive symptoms. For example, irritable or oppositional children may be difficult to parent and form positive relationships with, and this may affect the emotional wellbeing of parents. Third, parental depressive symptoms and offspring psychopathology may share a common aetiology.

Researchers have reported that parental depression/depressive symptoms predict offspring psychopathology (Fendrich *et al.* 1990; Davies & Windle, 1997; Lieb *et al.* 2002; Cummings *et al.* 2005), and offspring psychopathology predicts parental depression (Tamplin *et al.* 1998; Tan & Rey, 2005; Wilkinson *et al.* 2013). Longitudinal research shows these relationships can be reciprocal, with maternal depressive symptoms prospectively predicting the development of child problem behaviours, and vice versa (Gross *et al.* 2008*a*, *b*; Gross *et al.* 2009).

A major problem in interpreting research linking parent and child psychopathology is that in most cases parents and children are genetically related, so associations may be confounded by shared genes. Depression is under genetic influence in adulthood (Sullivan *et al.* 2000) and childhood (Rice *et al.* 2002; Thapar & Rice, 2006), so genetic factors could explain associations between parent and offspring emotional problems. Furthermore, genetic effects overlap across traits (Krueger *et al.* 2002; Kendler *et al.* 2003; McAdams *et al.* 2012; Bolhuis *et al.* 2014), so genetic commonalities could explain associations between parental depression and any heritable offspring outcome. Ultimately, intergenerational genetically informative data are required to properly assess the nature of the parent–child associations.

## Children-of-twins (CoT) studies

Studying twins and their offspring allows researchers to identify whether associations between parent and child phenotypes are genetic and/or environmental in nature (Fischer, 1971; Gottesman & Bertelsen, 1989; D'Onofrio *et al.* 2003; Silberg & Eaves, 2004; McAdams *et al.* 2014). The utility of a CoT sample has been described in detail elsewhere (McAdams *et al.* 2014), but is briefly described here. Because monozygotic (MZ) twins are genetically identical, their offspring are as genetically related to their parents’ co-twin as they are to their own parent. The relationship between a child and their parent's sibling is known as the avuncular relationship. In MZ twin families, if the parent–child correlation is greater than the avuncular correlation, then this indicates the presence of an association between parent and offspring phenotypes above and beyond familial confounding of genes and the extended family environment (e.g. the association is potentially attributable to parenting). If there is no difference then transmission is thought to be familial. The comparison between avuncular correlations in MZ *v.* dizygotic (DZ) families gives insight into the nature of familial effects. If the MZ avuncular correlation is larger, then genetic factors are implied.

Two CoT studies have investigated the relationship between parental depression and offspring depression and conduct problems (Silberg *et al.* 2010; Singh *et al.* 2011). Results from both indicated that the association between parent and child depression remained after accounting for familial confounds. One of the studies found that the association between parental depressive symptoms and adolescent conduct problems persisted after accounting for familial confounds (Silberg *et al.* 2010), whereas the other did not (Singh *et al.* 2011). Interestingly, neither study found evidence for genetic transmission running from parental depression to offspring depression, but both found evidence for genetic transmission from parental depression to conduct problems.

## Adoption studies

Studying children adopted at birth provides another geneticall -informed method of examining intergenerational associations – when adoptive parents are genetically unrelated to their child, parent–child correlations are not confounded by shared genotype. Conversely, biological parents of children adopted at birth provide only their genes (and intrauterine environment), so any correlation between their phenotype and that of their child's cannot be attributed to a (post-adoption) environmental effect (assuming any selective placement is controlled for). Researchers have reported that adoptive-parent depression correlates with offspring depression (Cadoret *et al.* 1985; Marmorstein *et al.* 2012) but not substance use (Marmorstein *et al.* 2012) in young adopted adults, and with offspring depression and disruptive behaviour in adopted adolescents (Tully *et al.* 2008). Using adoption data from the Early Growth and Development Study (EGDS), researchers have also shown that adoptive-parent depressive symptoms predicts child fussiness at age 18 months (Natsuaki *et al.* 2010), externalizing problems in toddlers aged 27 months (Pemberton *et al.* 2010), and internalizing and externalizing problems in early childhood (Kerr *et al.* 2013; Laurent *et al.* 2013*a*, *b*).

Using data from EGDS (Leve *et al.* 2013), researchers have shown that birth parent depressive symptoms have no main effect on the development of fussiness in toddlers (age 9–18 months; Natsuaki *et al.* 2010), a borderline significant effect on externalizing problems in toddlerhood (age 27 months; Pemberton *et al.* 2010), and predict externalizing but not internalizing problems in early childhood (ages 18–54 months; Kerr *et al.* 2013).

## The current study

Some CoT and adoption findings are beginning to converge in a coherent story. For example, associations between parental depressive symptoms and offspring emotional problems remain after controlling for familial confounds (shared genes and extended family environment) (Cadoret *et al.* 1985; Tully *et al.* 2008; Silberg *et al.* 2010; Singh *et al.* 2011; Marmorstein *et al.* 2012; Kerr *et al.* 2013; Laurent *et al.* 2013*a*). However, other results are inconsistent, with some studies finding an association between parental depression and offspring externalizing behaviours above and beyond familial confounds (Tully *et al.* 2008; Pemberton *et al.* 2010; Silberg *et al.* 2010; Kerr *et al.* 2013) where others do not (Singh *et al.* 2011; Marmorstein *et al.* 2012). Some findings stand out as unusual and in need of further examination. For example, parent depressive symptoms appear to have a non-significant genetic relationship with offspring internalizing problems but a significant association with offspring externalizing problems (Silberg *et al.* 2010; Singh *et al.* 2011; Kerr *et al.* 2013). Furthermore, no genetically informative study has yet tested for bidirectional effects between parental depressive symptoms and offspring psychopathology.

In the present study, we follow the advice of others (Rutter *et al.* 2001) in using complementary but distinct research designs to examine our research questions. We use a CoT sample and an adoption sample to address the following aims: (1) Evaluate the association between parental depressive symptoms and offspring internalizing/externalizing problems after accounting for familial confounds. (2) Identify the role of genetic transmission in these associations. (3) Evaluate these associations within a longitudinal framework allowing for the testing of bidirectional effects. Our CoT analysis will assist us in our first two aims, and our adoption sample in all three research aims.

Our CoT analysis will contribute to the literature by evaluating associations between parent depression and adolescent offspring internalizing/externalizing problems using a design employed for this purpose only twice previously. Our adoption study will examine longitudinal associations between birth parent depressive symptoms, adoptive-parent depressive symptoms, and offspring internalizing/externalizing problems in middle childhood. While previous studies have been cross-sectional or have focussed only on the impact of parental depression on offspring outcomes, our adoption study will be the first to examine bidirectional relationships between adoptive-parent depressive symptoms and offspring internalizing/externalizing problems. Further, it will be the first study examining this association in middle childhood.

## Ethical standards

The authors assert that all procedures contributing to this work comply with the ethical standards of the relevant national and institutional committees on human experimentation and with the Helsinki Declaration of 1975, as revised in 2008.

## Method

### CoT sample

Data were drawn from the Twin Offspring Study of Sweden (TOSS) and included 387 MZ and 489 DZ twin families (a same-sex twin pair, each with a spouse and an adolescent child). Twin offspring were selected so that cousins were the same sex and did not differ in age by more than 4 years. Thirty-seven percent of twin pairs and 52% of offspring were male. Mean ages were 15.7 years for offspring (s.d. = 2.4, range 11–22), 44.8 years for twins (s.d. = 4.9, range 32–60), and 45.5 years for spouses (s.d. = 5.4, range = 25–65). Further information on TOSS is given elsewhere (Neiderhiser & Lichtenstein, 2008).

### Adoption sample

Data were drawn from the EGDS, a sample of adopted children, and their birth parents and adoptive parents. EGDS comprises two cohorts followed from 3 months postpartum. We focus on the first (older) cohort, comprising 361 adoptive families: 361 sets of adoptive parents and 359 birth mothers. The mean age of birth mothers at the child's birth was 24.1 years (s.d. = 5.9, range = 14–43). The mean age of adoptive parents was 37.8 years (s.d. = 5.5, range = 22–54). In this study we focus primarily on assessments conducted when children were aged 4.5, 6 and 7 years. Forty-three per cent of the children were female. Further details on EGDS can be found elsewhere (Leve *et al.* 2013).

### Measures – CoT sample

*Parental depressive symptoms* were measured in twins using the self-report Center for Epidemiological Studies Depression scale (CES-D; Gatz *et al.* 1993), comprising 20 self-report items assessing the severity of depressive symptoms in the last week. The CES-D correlates with other depression measures and performs well in identifying depressed patients (Weissman *et al.* 1977). It has good internal consistency, and is a reliable measure for detecting depressive symptoms in Swedish samples (Gatz *et al.* 1993).

*Offspring internalizing and externalizing problems* were measured using the Child Behaviour Checklist (CBCL; Achenbach & Rescorla, 2001). Twins, spouses and offspring reported on offspring internalizing and externalizing problems, assessed with 32 and 30 items, respectively. Composite scales were created, taking the average of all reports. The CBCL is widely used and has been reported as a valid and reliable tool for assessing adolescent psychopathology (Dutra *et al.* 2004). Correlations between reporters ranged from 0.30 to 0.45 for offspring internalizing, and from 0.36 to 0.57 for offspring externalizing.

### Measures – adoption sample

*Parental depressive symptoms* were measured using the self-report Beck Depression Inventory (BDI; Beck *et al.* 1996). Items assessed the severity of depressive symptoms in the previous week. Adoptive parents were assessed when children were aged 4.5, 6, and 7 years. The depression score of the primary caregiver was used. In most cases this was the adoptive mother. In same-sex families the parent who adopted the role of primary caregiver was used (6 families of 2 adoptive mothers and 10 families of 2 adoptive fathers). Birth-mother symptoms were assessed at 4 months, 18 months and 4.5 years post-adoption. In EGDS 20 BDI items were used (suicidal ideation was excluded from the questionnaire). The BDI is widely reported as a valid and reliable measure of depressive symptoms (Beck *et al.* 1988).

*Child internalizing and externalizing problems* were measured using the CBCL. Both adoptive parents were asked to report on their child's behaviour when children were aged 4.5, 6, and 7 years. Composite scales were created at each age, taking the average of parent reports. Correlations between parents ranged from 0.40 to 0.45 for offspring internalizing, and from 0.47 to 0.57 for externalizing.

*Control variables:* Obstetric complications and adoption openness (contact between birth and adoptive families) were controlled for. *Obstetric complications* were measured using a pregnancy screener that birth mothers completed (assessing weight change, blood pressure, vitamin use, medications, laboratory tests, due/birth dates, timing/frequency of doctor visits, and symptoms of illnesses), and a pregnancy history calendar, wherein mothers reported perinatal substance use and psychopathology. Further details can be found elsewhere (Marceau *et al.* 2013). *Adoption openness* was measured by asking adoptive parents how much contact they had with birth parents at every wave of data collection. Birth parents were asked to report how much contact they had with adoptive parents at 4 months, 18 months and 4.5 years postpartum. The measure used in the present study is a composite of birth-mother and adoptive-parent reports of adoption openness at each wave (see Ge *et al.* 2008).

### Analyses: children of twins

Prior to analyses residuals were taken to control for twin sex and age†. All variables were log-transformed to correct for skew. We fitted structural equation models using maximum likelihood estimation in the programme OpenMx (Boker *et al.* 2011). Models allowed us to quantify the effects of additive genetic (A), common environmental (C; non-genetic effects that make members of a family similar to one another) and non-shared environmental effects (E; environmental effects that make members of a family different to one another) on parental depressive symptoms. By comparing the magnitude of MZ twin correlations (attributable to A + C) to DZ twin correlations [attributable to (0.5 × A) + C], genetic and environmental influences were estimated. Comparing MZ and DZ avuncular correlations to parent–child correlations allowed for estimation of genetic and environmental intergenerational pathways. Comparing correlations between cousins from MZ and DZ families allowed for the estimation of genetic and non-shared environmental effects on offspring phenotype. The full CoT model is included in the Supplementary online material (Supplementary Fig. S1), along with matrix specifications (Supplementary Table S1). Of note, and in contrast to more typical multivariate twin models, our model includes a direct ‘phenotypic transmission’ pathway between parental phenotype and offspring phenotype. This path is designed to capture covariance between parent and offspring phenotypes not attributable solely to direct genetic transmission – effects associated with exposure to parental phenotype. Purely genetic transmission is captured by a path linking parent and child genetic factors. The significance of pathways were tested by creating sub-models in which paths were fixed to zero. *χ*^2^ difference tests and Akaike's Information Criterion were used to assess whether sub-models yielded a significantly worse fit to the data than the full model.

### Analyses: adoption sample

Using maximum likelihood estimation in Mplus 6.11 (Muthén & Muthén, 1998–2010) we fitted an autoregressive cross-lagged model to the adoption data, with the addition of birth-mother depressive symptoms as a measure of genetic risk. This model was designed as a parsimonious method of assessing whether longitudinal phenotypic associations between adoptive parent and child remained after accounting for ‘genetic risk’. We followed Pemberton *et al.* (2010) by defining birth-mother depressive symptoms as a latent variable comprising self-reported depressive symptoms at 4, 18, and 54 months postpartum. The resultant variable indexes stable depression, and reduces measurement error by minimizing the impact of time-point specific variations in symptoms. It is thus well suited to index genetic risk for depression since prior research shows that persistent, stable depression is under greater genetic influence than less stable or temporary forms of depression (Kendler *et al.* 2001). This latent variable was included as a predictor of child internalizing/externalizing problems at all ages.

Obstetric complications were included as a covariate in associations between birth-mother depressive symptoms and child internalizing/externalizing symptoms at age 4.5 years (the earliest measurement in our model). Openness of adoption was included as a covariate in associations involving child internalizing/externalizing symptoms.

## Results: children of twins

Descriptive statistics and phenotypic correlations are included in the Supplementary material (Supplementary Tables S2 and S3).

### Twin correlations

Comparing twin correlations in Table 1, the MZ twin correlation of 0.35 was larger than the DZ twin correlation of 0.14, indicating that parental depression was heritable. Point estimates suggested correlations between cousins in MZ families were larger than those in DZ families, although confidence intervals suggested differences were not statistically significant (0.16 *v.* 0.10 and 0.22 *v.* 0.14, for internalizing and externalizing, respectively). Parent–child correlations were higher than MZ avuncular correlations (0.26 *v.* 0.07 and 0.18 *v.* 0.09), suggesting intergenerational associations may not be wholly attributable to familial confounds. Most avuncular correlations were non-significant, and no MZ avuncular correlations were significantly larger than DZ avuncular correlations, suggesting genetic factors do not play a primary role in explaining intergenerational correlations.

**Table 1.** Intraclass correlations for the relationships between twin parental depression and offspring internalizing/externalizing problems (95% confidence intervalsin parentheses)

	MZ twin families			DZ twin families		
	Twin 1	Twin 2	Offspring 1	Twin 1	Twin 2	Offspring 1
Parental depression and offspring internalizing						
Twin 2	0.35 (0.26–0.43)			0.14 (0.04–0.23)		
Offspring 1	0.26 (0.21–0.30)	0.07 (0.00–0.14)		0.26 (0.21–0.30)	0.04 (−0.03–0.10)	
Offspring 2	0.07 (0.00–0.14)	0.26 (0.21–0.30)	0.16 (0.06–0.25)	0.04 (−00.03–0.10)	0.26 (0.21–0.30)	0.10 (0.01–0.19)
Parental depression and offspring externalizing						
Twin 2	0.35 (0.26–0.43)			0.13 (0.04–0.22)		
Offspring 1	0.18 (0.14–0.23)	0.09 (0.02–0.16)		0.18 (0.14–0.23)	0.04 (−0.03–0.10)	
Offspring 2	0.09 (0.02–0.16)	0.18 (0.14–0.23)	0.22 (0.11–0.31)	0.04 (−0.03–0.10)	0.18 (0.14–0.23)	0.14 (0.05–0.22)

Twin 1 is the parent of child 1. Twin 2 is the parent of child 2. Correlations are taken from constrained saturated models in which variances and means were constrained across twin order and zygosity and parent–child covariances were constrained across zygosity.

### Structural equation modelling

**Fig. 1.**
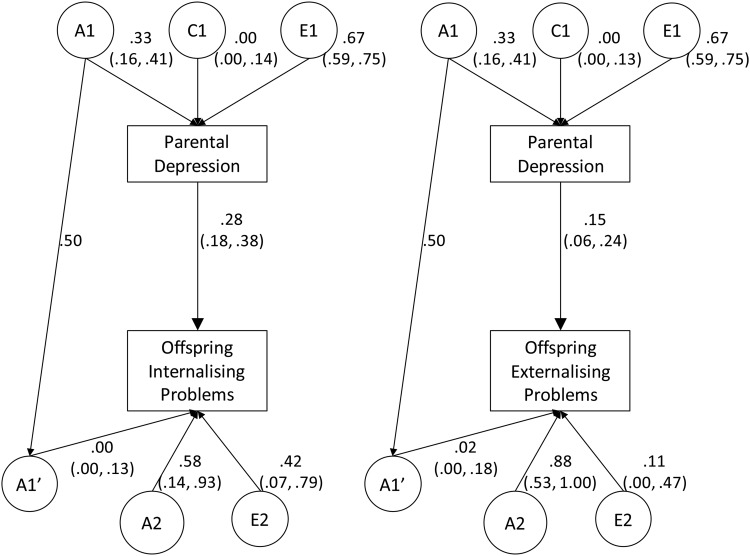
Path diagrams showing the relationship between parental depression and offspring internalizing/externalizing problems. Path estimates are taken from the full (unconstrained) models in which all parameters are freely estimated. A1, Additive genetic effects on parental depression; C1, shared-environmental effects on parental depression; E1, non-shared environmental effects on parental depression; A1′, genetic effects common to parental depression and offspring phenotype; A2, familial effects specific to offspring phenotype; E2, non-shared environmental effects on offspring phenotype. Note that the pathway between A1 and A1′ is fixed to 0.50 because parents and children share 50% of their genome.

Path diagrams are presented in Fig. 1. Model fitting (Supplementary Table S4) showed that dropping the phenotypic association from the internalizing model (*p*: estimated at 0.28 in Fig. 1) significantly worsened model fit, but dropping the genetic pathway (A1’: estimated at 0.00) did not. Similarly, dropping the phenotypic association from the externalizing model (*p*: estimated at 0.15) led to a significantly worse model fit, but eliminating the genetic pathway (A1’: estimated at 0.02) did not. These findings demonstrate that associations between parental depression and offspring internalizing and externalizing problems were not attributable to genetic transmission.

## Results: adoption study

Descriptive statistics and correlations are included in Supplementary Tables S5 and S6.

### Structural equation modelling

**Fig. 2.**
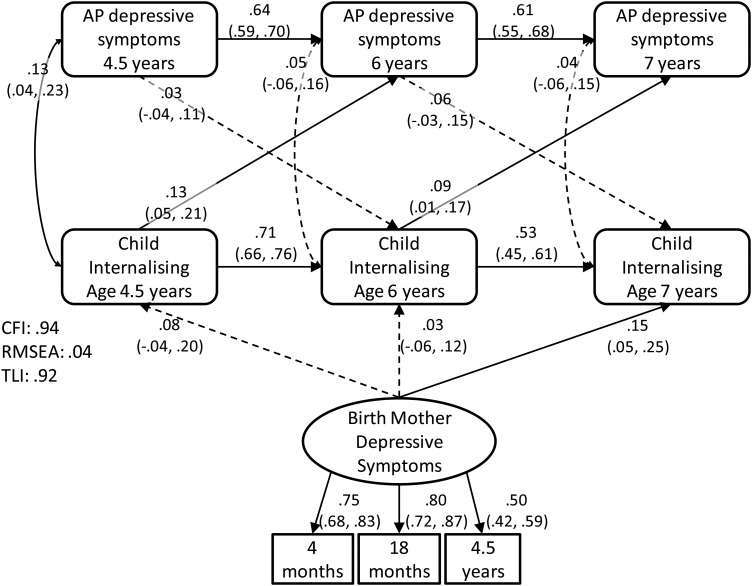
Structural equation model showing the relationship between parental depressive symptoms and offspring internalizing problems in the EGDS sample (95% confidence intervals). Parameter estimates are all standardized. Significant pathways are represented with solid lines, non-significant pathways are dashed. This is the full (unconstrained) model in which all parameters are freely estimated. AP, Adoptive parent. For adoptive-parent depressive symptoms at 6 years, *R*^2^ = 0.45, *p* < 0.001. For adoptive-parent depressive symptoms at 7 years, *R*^2^ = 0.41, *p* < 0.001. For child internalizing at 6 years *R*^2^ = 0.52, *p* < 0.001. For child internalizing at 7 years *R*^2^ = 0.34, *p* < 0.001.

**Fig. 3.**
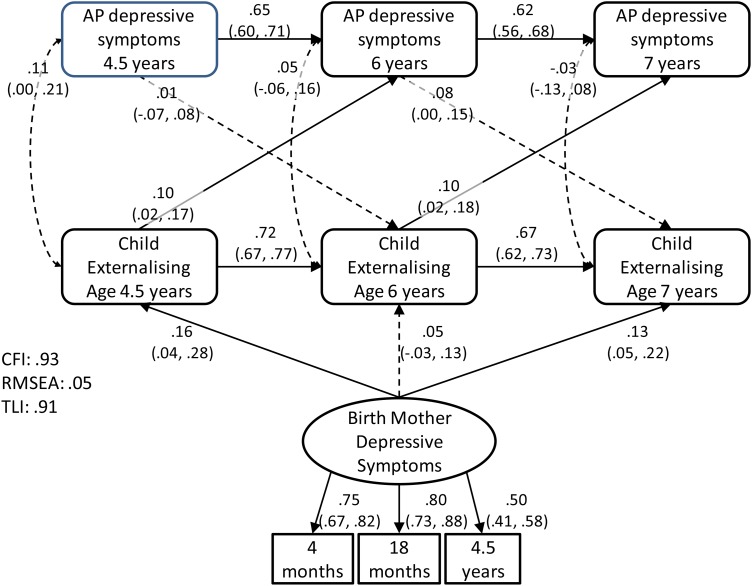
Structural equation model showing the relationship between parental depressive symtoms and offspring externalizing problems in the EGDS sample (95% confidence intervals). Parameter estimates are all standardized. Significant pathways are represented with solid lines, non-significant pathways are dashed. This is the full (unconstrained) model in which all parameters are freely estimated. AP, Adoptive parent. For adoptive-parent depressive symptoms at 6 years, *R*^2^ = 0.45, *p* < 0.001. For adoptive-parent depressive symptoms at 7 years, *R*^2^ = 0.41, *p* < 0.001. For child externalizing at 6 years *R*^2^ = 0.53, *p* < 0.001. For child externalizing at 7 years *R*^2^ = 0.53, *p* < 0.001.

Models for internalizing (Fig. 2) and externalizing problems (Fig. 3) tell similar stories. Cross-lagged paths indicated that child internalizing and externalizing problems were predictive of later adoptive-parent depressive symptoms (range 0.09–0.13). Of note, paths from adoptive-parent depressive symptoms to subsequent child internalizing and externalizing problems were not significant (range 0.01–0.08). Birth-mother depressive symptoms predicted child internalizing at age 7 years but not at 4.5 or 6 years, and predicted child externalizing at 4.5 and 7 years, but not at 6 years. Although it may seem unusual that birth-mother depressive symptoms predicted externalizing problems at 4.5 and 7 years but not 6 years, we attribute this to the short time lapse between 4.5 and 6 years, combined with the high continuity in externalizing between waves (0.72). Together this probably left little genetic variance in externalizing behaviour to account for at age 6 years above that already accounted for at age 4.5 years.

## Discussion

We set out to establish the nature of the association between parental depressive symptoms and offspring internalizing and externalizing problems using the TOSS CoT sample, and the EGDS adoption sample. Results from both analyses demonstrated that phenotypic associations remained after accounting for genetic transmission, suggesting associations result from environmental influences. These findings align with previous similar studies (Cadoret *et al.* 1985; Tully *et al.* 2008; Natsuaki *et al.* 2010; Pemberton *et al.* 2010; Silberg *et al.* 2010; Singh *et al.* 2011; Marmorstein *et al.* 2012; Kerr *et al.* 2013; Laurent *et al.* 2013*a*, *b*). We are aware of only one published finding to contradict ours – a CoT analysis, wherein it was found that the association between parental depression and conduct problems was not significant after accounting for genetic transmission (Singh *et al.* 2011). This particular study used diagnostic criteria to define depression and conduct problems in young adults, where our study, and those of others whose findings concur with our own, assessed continuous measures of subclinical symptoms in adolescents. It is possible that the difference in measurement approach or age explains the difference in results.

Where previous genetically informative studies have not considered the possibility of child-to-parent effects, our analysis of the EGDS dataset was designed to assess bidirectional effects between parent and child. Intriguingly, models indicated that child internalizing and externalizing symptoms predicted subsequent parental depressive symptoms, but the reverse was not true. This is what would be expected if children's emotional and behavioural difficulties affect the emotional wellbeing of their parents. It should be noted, however, that confidence intervals on the parent–child and child–parent paths overlapped. As such we cannot conclude that child-to-parent effects are significantly larger than parent-to-child effects. Regardless, ours is the first genetically informed study to show that child internalizing and externalizing problems prospectively predict parental depressive symptoms.

### Genetic associations between parental depressive symptoms and offspring internalizing and externalizing problems

While results from both studies demonstrated that associations between parental depressive symptoms and offspring internalizing/externalizing persisted after controlling for genetic transmission, findings relating to genetic transmission were not entirely consistent across samples. TOSS results support previous CoT studies in finding no genetic transmission between parental depressive symptoms and offspring internalizing (Silberg *et al.* 2010; Singh *et al.* 2011). Our EGDS findings are the first of their kind however, showing a significant effect of birth-parent depressive symptoms on offspring internalizing problems. Previous CoT and adoption studies have found no such association. It is possible that age explains our findings, or more precisely, the age gap between parent and child. Birth-mother depression in EGDS was defined as depressive symptoms in young adults (birth mothers), and this predicted offspring internalizing problems at age 7 years. In previous studies, age gaps were larger, with parents being in middle age and offspring in adolescence (Cadoret *et al.* 1985; Tully *et al.* 2008; Silberg *et al.* 2010; Singh *et al.* 2011; Marmorstein *et al.* 2012), or birth parents being young adults and their children in early childhood (Natsuaki *et al.* 2010; Pemberton *et al.* 2010; Kerr *et al.* 2013). It is known that genetic factors involved in depression are not static and change over time, so it may be that genetic overlap between parent and offspring emotional problems decreases as the age gap between them increases.

On a related note, the lack of evidence in support of genetic transmission in our TOSS analysis and in other CoT analyses should not be viewed as contradicting the notion that familial transmission of depression is at least partially genetic. Rather, we show that the association between *concurrent* parental depressive symptoms and adolescent offspring internalizing problems is not attributable to genetic transmission. The absence of such associations may be indicative of different genes influencing depression during different developmental periods. If we were to measure depressive symptoms at the same age in parents and offspring, we would expect evidence for genetic transmission.

Ours is the first CoT study to report no genetic transmission between parental depressive symptoms and adolescent externalizing problems. However, our EGDS findings conform to previous CoT (Silberg *et al.* 2010; Singh *et al.* 2011) and adoption (Pemberton *et al.* 2010; Kerr *et al.* 2013) studies in demonstrating an association between genetic risk for depressive symptoms and offspring externalizing problems. It is intriguing that the evidence for genetic transmission from parent depressive symptoms to offspring externalizing problems is more consistent across studies than that from parent depression to offspring internalizing problems. It is possible that childhood externalizing problems may have more genes in common with adult depression than does childhood internalizing problems. Symptomatically depression would appear to have more overlap with internalizing than externalizing so this finding is not intuitive. It would be interesting to examine whether child externalizing problems and adult depression have greater genetic overlap than child internalizing and adult depression in longitudinal twin studies.

The genetic overlap between child externalizing problems and adult depressive symptoms is compatible with the inclusion of ‘irritable mood’ as a core symptom of DSM-5 depression (APA, 2013) only when the onset is during childhood. Of note, irritability, one facet of externalizing behaviour, has been shown to have greater phenotypic and genetic links with depression than with delinquency (Stringaris *et al.* 2012). Thus it may be that during childhood, genetic risk for depression manifests as irritability, a trait more often captured by measures of externalizing than internalizing problems.

### Comparing the results from EGDS and TOSS

We found evidence for genetic effects in EGDS but not TOSS. One reason for this could be that mothers whose children are adopted at birth are at greater genetic risk for depression than are twin mothers, and therefore pass on greater genetic risk to their children. Another possibility relates to our use of a latent factor to index genetic risk in EGDS. This factor was defined by multiple assessments carried out over several years, so captured variance common to depressive symptoms at 3 time points. Persistent depression has been reported as being under greater genetic influence than single episodes of depression (Kendler *et al.* 2001), and longitudinal twin studies demonstrate that continuity in depression is predominantly attributable to genetic effects (Lau & Eley, 2006; Kendler *et al.* 2008). As such, our use of longitudinal data to create a latent proxy measure of genetic risk in EGDS may explain why we find genetic effects in EGDS but not TOSS.

It is worth noting that alternative approaches to the TOSS CoT data may have detected genetic overlap between parental depression and offspring internalizing/externalizing problems. For example, we also explored applying standard multivariate twin models to our data (i.e. a Cholesky decomposition and a correlated factors solution) wherein offspring internalizing/externalizing problems were modelled as a parental phenotype. These models suggested that some of the correlation could be attributed to genetic overlap. As such, our findings do not necessarily mean that there is no overlap in the genes involved in parent depressive symptoms and offspring internalizing/externalizing problems. Rather, they suggest that the intergenerational association is best conceptualised as environmental in nature (i.e. attributable to exposure).

### Limitations

In the present study we used CoT and adoption data to complement one another; each approach providing distinct techniques through which to assess the nature of associations between parental depression and offspring internalizing/externalizing problems (Rutter *et al.* 2001). However, despite assessing the same phenotypes using overlapping measures, TOSS and EGDS were not perfectly complementary to one another. Specifically, children were in different developmental periods in each (childhood in EGDS, adolescence in TOSS). However, in spite of this, the two studies concur in many of their findings. Specifically, phenotypic associations persisted after controlling for genetic overlap. As a result there does not appear to be a reason to assume that the age difference between TOSS and EGDS offspring has unduly impacted our results.

Our analyses were underpowered to explore sex differences. However, some researchers have previously reported that sex differences may exist in the association between parental depression and offspring psychopathology (e.g. Davies & Windle, 1997). In the future it is hoped that genetically informative studies will be large enough to have the power to examine sex differences in pathways from paternal/maternal depression to male/female offspring.

In TOSS, both parents and the child reported on adolescent externalizing and internalizing problems, but in EGDS only parents reported on child adjustment. While the use of self-report is not practicable in child samples, it is possible that the different approaches to measurement may have contributed to differences in results. Another limitation is that both studies involved the use of normative samples, so it is unclear to what extent findings generalise to clinical populations.

## Conclusions

Limitations notwithstanding, results of the present study suggest that phenotypic relationships between parental depression and offspring emotional and behavioural problems are significant above and beyond potential genetic confounding. Where prior studies have not tested for bidirectional effects, our EGDS findings suggest that the residual phenotypic association (that remaining after accounting for genetic overlap) should not be assumed to be inherently parent-to-child in nature, but involves child-to-parent effects. If the association between parental depression and offspring emotional and behavioural problems is not wholly genetic in nature, and is bidirectional, then interventions aimed at reducing parental depression and/or child emotional and behavioural problems would do well to target the parent–child dyad together.

## Footnotes

† In response to a reviewer's request we also recomputed all analyses with data that had been regressed on child sex and age as well as twin sex and age. Resultant models changed very little and conclusions remained the same whether regressing on child sex and age or not.

## Supplementary material

For supplementary material accompanying this paper visit http://dx.doi.org/10.1017/S0033291715000501. click here to view supplementary material
