# Supplementary material for: The relationship between parental depressive symptoms and offspring psychopathology: evidence from a children-of-twins study and an adoption study
Source: Psychol Med. 2015 May 21;45(12):2583–94. doi: 10.1017/S0033291715000501 (PMC4523449; doi:10.1017/S0033291715000501)
Supplement: Supplementary file 1 [file S0033291715000501sup001.docx]

**SUPPLEMENTARY MATERIAL**

Figure S1. Full children-of-twins structural equation model showing correlations for monozygotic and dizygotic twin families

A1=Additive genetic effects on parental depression; C1= shared-environmental effects on parental depression; E1=nonshared environmental effects on parental depression; A1’=genetic effects common to parental depression and offspring phenotype; A2=familial effects specific to offspring phenotype; E2=nonshared environmental effects on offspring phenotype. NB the pathway between A1 and A1’ is fixed to .50 because parents and children share 50% of their genome.

Table S1. Matrix Specification of the Children-of-Twins Model

| Variance-covariance matrix for monozygotic twins | | | | |
| --- | --- | --- | --- | --- |
|  | Parent (Twin) 1 | Parent (Twin) 2 | Child 1 | Child 2 |
| Parent 1 | a1^2^ + c1^2^ + e1^2^ |  |  |  |
| Parent 2 | a1^2^ + c1^2^ | a1^2^ + c1^2^ + e1^2^ |  |  |
| Child 1 | p+.5 a1’ a1 | p a1^2^+p c1^2^+.5 a1’ a1 | a2^2^ + a1’^2^ + e2^2^ |  |
| Child 2 | p a1^2^+p c1^2^+  .5 a1’ a1 | p+.5 a1’a1 | .25 a2^2^ + p^2^ c1^2^ + p^2^ a1^2^ + .25 a1’^2^ | a2^2^ + a1’^2^ + e2^2^ |
| Variance-covariance matrix for dizygotic twins | | | | |
|  | Parent (Twin) 1 | Parent (Twin) 2 | Child 1 | Child 2 |
| Parent 1 | a1^2^ + c1^2^ + e1^2^ |  |  |  |
| Parent 2 | .5 a1^2^ + c1^2^ | a1^2^ + c1^2^ + e1^2^ |  |  |
| Child 1 | p+.5 a1’ a1 | .5 p a1^2^+p c1^2^+  .25 a1’ a1 | a2^2^ + a1’^2^ + e2^2^ |  |
| Child 2 | .5 p a1^2^+p c1^2^+  .25 a1’ a1 | p+.5 a1’a1 | .125 a2^2^ + p^2^ c1^2^ + .5 p^2^ a1^2^ + .125a1’^2^ | a2^2^ + a1’^2^ + e2^2^ |

Table S2. Descriptive Statistics for the TOSS sample

|  | Mean | Standard Deviation |
| --- | --- | --- |
| Twin Depression (CES-D) | 9.97 | 8.09 |
| Offspring internalising (CBCL) | 5.41 | 3.83 |
| Offspring externalising (CBCL) | 7.19 | 4.30 |

Levels of depression were equivalent in twins and their spouses (*t*(1718) = 0.18, *p*=.85).

Table S3. Phenotypic associations in the TOSS sample

|  | Twin Depression | Offspring Internalising (CBCL) |
| --- | --- | --- |
| Offspring internalising | .24 |  |
| Offspring externalising | .17 | .44 |

All are significant to the p < .001 level.

Table S4. Model Fitting: Structural models for parental depression and offspring internalising and externalising problems

| Internalising | | -2LL | AIC | DF | χ^2^ | p |
| --- | --- | --- | --- | --- | --- | --- |
|  | Saturated model | 9620.58 | 2750.58 | 3435 |  |  |
|  | Full model | 9635.29 | 2727.29 | 3454 |  |  |
|  | **Phenotypic Transmission (No genetic path)*** | **9365.49** | **2725.49** | **3455** | **0.20 (1)** | **.65** |
|  | Genetic Transmission (No phenotypic path)** | 9671.04 | 2761.04 | 3455 | 35.75 (1) | <.001 |
| Externalising | |  |  |  |  |  |
|  | Saturated model | 9664.63 | 2794.63 | 3435 |  |  |
|  | Full model | 9680.93 | 2772.93 | 3454 |  |  |
|  | **Phenotypic Transmission (No genetic path)*** | **9681.64** | **2771.64** | **3455** | **0.72 (1)** | **.40** |
|  | Genetic Transmission (No phenotypic path)** | 9690.97 | 2780.97 | 3455 | 10.04 (1) | <.001 |

NB: best fitting models are highlighted in bold; * in this model A1’ was fixed to zero; **in these models path p was fixed to zero.

Table S5. Descriptive Statistics for the EGDS sample

|  | Mean | SD |
| --- | --- | --- |
| AP Depressive symptoms (BDI), 4.5 years | 4.48 | 4.37 |
| AP Depressive symptoms (BDI), 6 years | 5.12 | 4.51 |
| AP Depressive symptoms (BDI), 7 years | 5.20 | 4.66 |
| BM Depressive symptoms (BDI), 4 months | 11.13 | 9.80 |
| BM Depressive symptoms (BDI), 18 months | 11.17 | 9.41 |
| BM Depressive symptoms (BDI), 4.5 years | 7.07 | 7.47 |
| Child internalising (CBCL), 4.5 years | 7.24 | 4.83 |
| Child internalising (CBCL), 6 years | 6.69 | 4.82 |
| Child internalising (CBCL), 7 years | 4.02 | 3.41 |
| Child externalising (CBCL), 4.5 years | 12.14 | 6.02 |
| Child externalising (CBCL), 6 years | 9.74 | 6.18 |
| Child externalising (CBCL), 7 years | 6.84 | 5.15 |

AP=Adoptive parent. BM=Birth mother

Table S5: Adoptive parent depressive symptoms increased in severity slightly over time, with the increase from child age 4.5 and 6 years (*t*(269) = 3.01, *p* < .01). Birth mother depressive symptoms were considerably higher than adoptive parent depressive symptoms, with significant differences between even the lowest birth mother depressive symptom scores (4.5 years post-adoption) and the highest adoptive parent depressive symptom scores (child age 7 years) (*t*(248) = 3.13, *p* < .01). Adoptee internalising symptoms significantly reduced from ages 4.5 to 6 years (*t(232) = 3.30, p* < .001) and ages 6 to 7 years (*t(231) = 9.00, p* < .001). Similarly child externalising symptoms significantly reduced between ages 4.5 and 6 years (*t(232) = 9.09, p* < .001) and ages 6 to 7 years (*t(231) = 9.52, p* < .001).

Table S6. Pairwise correlations between variables in the EGDS sample

|  | 1. | 2 | 3 | 4 | 5 | 6 | 7 | 8 | 9 | 10 | 11 |
| --- | --- | --- | --- | --- | --- | --- | --- | --- | --- | --- | --- |
| 1. AP Depression, 4.5 y |  |  |  |  |  |  |  |  |  |  |  |
| 2. AP Depression, 6 y | .65* |  |  |  |  |  |  |  |  |  |  |
| 3. AP Depression, 7 y | .58* | .64* |  |  |  |  |  |  |  |  |  |
| 4. BM Depression, 4m | -.09 | -.10 | -.03 |  |  |  |  |  |  |  |  |
| 5. BM Depression, 18m | -.09 | -.06 | -.04 | .66* |  |  |  |  |  |  |  |
| 6. BM Depression, 4.5 y | -.17* | -.08 | -.11 | .40* | .41* |  |  |  |  |  |  |
| 7. Child internalising, 4.5y | .09 | .15* | .11 | .10 | .03 | -.06 |  |  |  |  |  |
| 8. Child internalising, 6y | .07 | .14* | .17* | .07 | .06 | -.09 | .70* |  |  |  |  |
| 9. Child internalising, 7y | .07 | .10 | .12 | .11 | .10 | .11 | .44* | .51* |  |  |  |
| 10. Child externalising 4.5y | .04 | .10 | .14* | .15* | .11 | .03 | .60* | .45* | .42* |  |  |
| 11. Child externalising, 6y | .04 | .13* | .21* | .10 | .13* | -.03 | .40* | .66* | .37* | .75* |  |
| 12. Child externalising, 7y | .04 | .11 | .15* | .14* | .22* | .04 | .30* | .38* | .55* | .68* | .71* |

*significant to at least the *p*<.05 level. AP=Adoptive parent. BM=Birth mother

Cross-time correlations within construct are highlighted in light grey. Correlations between parent and child measures are highlighted in dark grey.

Pairwise correlations between study variables are presented in Table 3. Cross-wave correlations highlighted in light grey demonstrate moderate to strong continuity in all variables over time. Correlations between parents and children are highlighted in dark grey. Correlations between adoptive parent depression and offspring internalising (top left dark grey quadrant) and externalising (bottom left dark grey quadrant) were small in magnitude, ranging from .07 to .17 for internalising and .04 to .21 for externalising. Significant correlations were found between child internalising/externalising problems and concurrent or future parental depression. Birth mother depression was significantly correlated with externalising behaviour in children (in 4 out of 9 instances) but not internalising problems (top right and bottom right dark grey quadrants).
